# Combined Mitral Valve Replacement and Ravitch Procedures in a Patient with Previous Pneumonectomy: Case Report and Review of the Literature

**DOI:** 10.21470/1678-9741-2018-0055

**Published:** 2018

**Authors:** Ilyas Kayacioglu, Ahmet Can Topcu, Kamile Ozeren, Yasin Ozden, Ahmet Bolukcu, Mehmet Yildirim

**Affiliations:** 1 Department of Cardiovascular Surgery, Dr. Siyami Ersek Thoracic and Cardiovascular Surgery Training and Research Hospital, Istanbul, Turkey.; 2 Department of Thoracic Surgery, Dr. Siyami Ersek Thoracic and Cardiovascular Surgery Training and Research Hospital, Istanbul, Turkey.

**Keywords:** Chest Wall/surgery, Heart Valve Prosthesis Implantation, Mitral Valve/surgery, Funnel Chest, Pneumonectomy

## Abstract

**Introduction:**

Significant anatomical and functional changes occur following pneumonectomy. Mediastinal structures displace toward the side of the resected lung, pulmonary reserve is reduced. Owing to these changes, surgical access to heart and great vessels becomes challenging, and there is increased risk of postoperative pulmonary complications.

**Methods:**

We performed a mitral valve replacement combined with a Ravitch procedure in a young female with previous left pneumonectomy and pectus excavatum.

**Results:**

She was discharged on postoperative day 9 and remains symptom-free 3 months after surgery.

**Conclusion:**

Thorough preoperative evaluation and intensive respiratory physiotherapy are essential before performing cardiac operations on patients with previous pneumonectomy.

**Table:** 

Abbreviations, acronyms & symbols	
**CABG**	**= Coronary artery bypass grafting**
**CPB**	**= Cardiopulmonary bypass**
**CT**	**= Computed tomography**
**Cx**	**= Circumflex**
**FEV_1_**	**= Forced expiratory volume in 1^st^ second**
**FVC**	**= Forced vital capacity**
**LAD**	**= Left anterior descending**
**LITA**	**= Left internal thoracic artery**
**MRI**	**= Magnetic resonance imaging**

## INTRODUCTION

Significant anatomical and functional changes occur following pneumonectomy. Mediastinal structures displace toward the side of the resected lung, pulmonary reserve is reduced, and the remaining lung compensatorily enlarges and herniates over the midline with elevation of the diaphragm^[1,2]^. Owing to these changes, surgical access to the heart and great vessels becomes challenging, and there is an increased risk of postoperative pulmonary complications.

## CASE REPORT

A 24-year-old female patient presented to our clinic with dyspnea. She had undergone a left pneumonectomy for advanced and complicated bronchiectasis 10 years ago.

### Clinical Findings

**Fig. 1:**
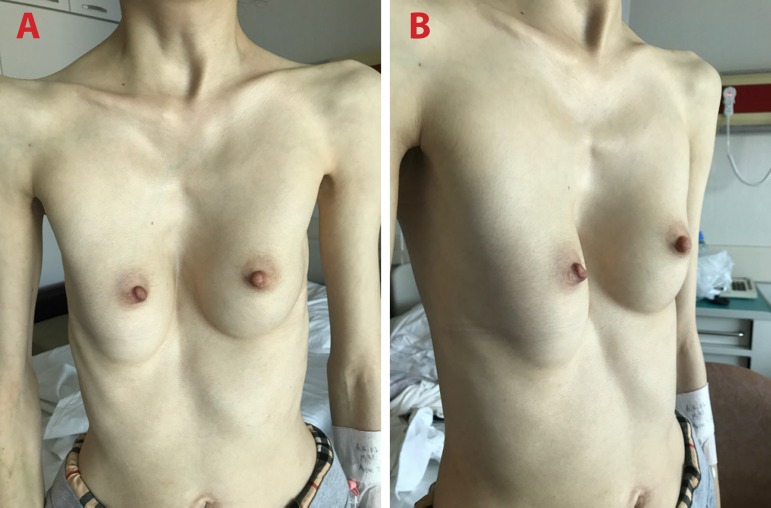
Marfanoid habitus and pectus excavatum. A) front view; B) side view.

She had marfanoid habitus, pectus excavatum, scoliosis, and a grade 4, pansystolic, high-pitched, blowing murmur best heard at the right sternal border (Figures 1A and B).

### Diagnostic Assessment

**Fig. 2:**
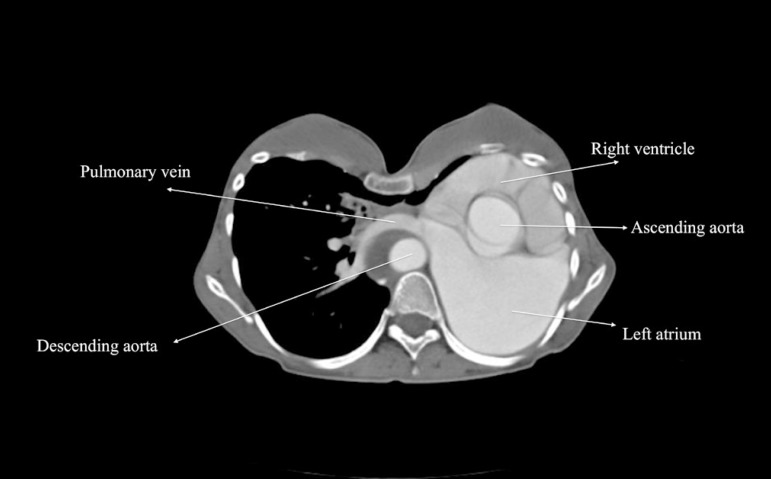
Contrast-enhanced computed tomography scan.

Transthoracic echocardiogram revealed severe mitral regurgitation due to myxomatous mitral valve with bileaflet prolapse and chordal elongation, secondary pulmonary hypertension, and tricuspid regurgitation with a dilated right atrium. Her ejection fraction was 35%, left ventricle end-diastolic diameter was 72 mm, and end-systolic diameter was 59 mm. She also had a borderline ascending aortic aneurysm measuring 40 mm in diameter. Pulmonary function test demonstrated reduced forced vital capacity (FVC), 1.11 L (31.7% of predicted), and reduced forced expiratory volume in 1^st^ second (FEV_1_), 1.05 L (34.6% of predicted). A contrast-enhanced computed tomography (CT) scan was performed to examine the mediastinal structures and alternative cannulation sites (Figure 2). Heart and great vessels were displaced to the left, and the right lung was enlarged and crossing the midline, anterior to the heart. The proxymal ascending aorta was 40 mm in diameter. Additionally, a chronic type B aortic dissection was present. CT scan revealed that the ascending aorta and the superior and inferior venae cavae were suitable for cannulation.

### Therapeutic Intervention

The patient received intensive chest physiotherapy before surgery to reduce postoperative pulmonary complications.

**Fig. 3:**
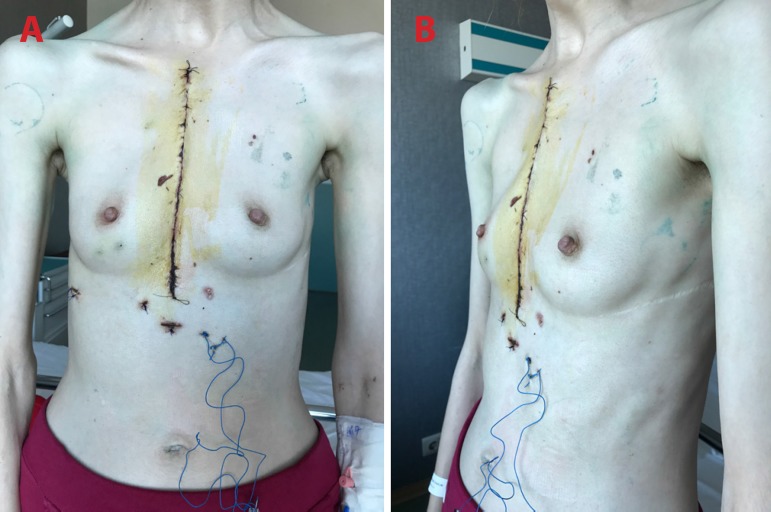
Early postoperative results. A) front view; B) side view.

A vertical midline incision on skin, subcutaneous tissues, and pectoralis fascia was made over the sternum. Following elevation of pectoralis muscles from the anterior chest wall, a median sternotomy was performed. Costal cartilages of the 3^rd^ to 8^th^ ribs were removed. The right lung was retracted from the midline. Cardiopulmonary bypass (CPB) was initiated via ascending aortic and bicaval cannulation, and cardiac arrest was obtained. We did not use topical cardiac hypothermia to prevent phrenic nerve injury. Both atria were relatively easy to expose due to leftward shift and rotation of the heart. A mitral valve replacement and a tricuspid ring annuloplasty was performed using biatrial approach. CPB was terminated. A bar was placed behind the sternum and fixed to the pectoralis muscle fibers bilaterally. After completion of the Ravitch procedure, the sternum was closed. The patient was transferred to a dedicated cardiac surgery intensive care unit and she was successfully extubated at the postoperative 6^th^ hour. Her recovery was uneventful and she was discharged on postoperative day 9 (Figures 3A and B).

### Follow-up and Outcomes

**Fig. 4:**
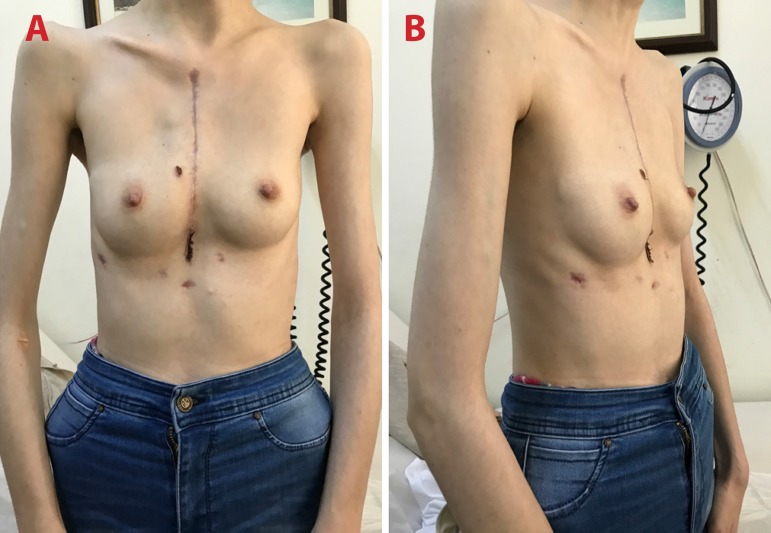
Late postoperative results. A) front view; B) side view.

The patient remains symptom-free 3 months after surgery and she is scheduled to have a bar removal 3 months later (Figures 4A and B).

**Fig. 5:**
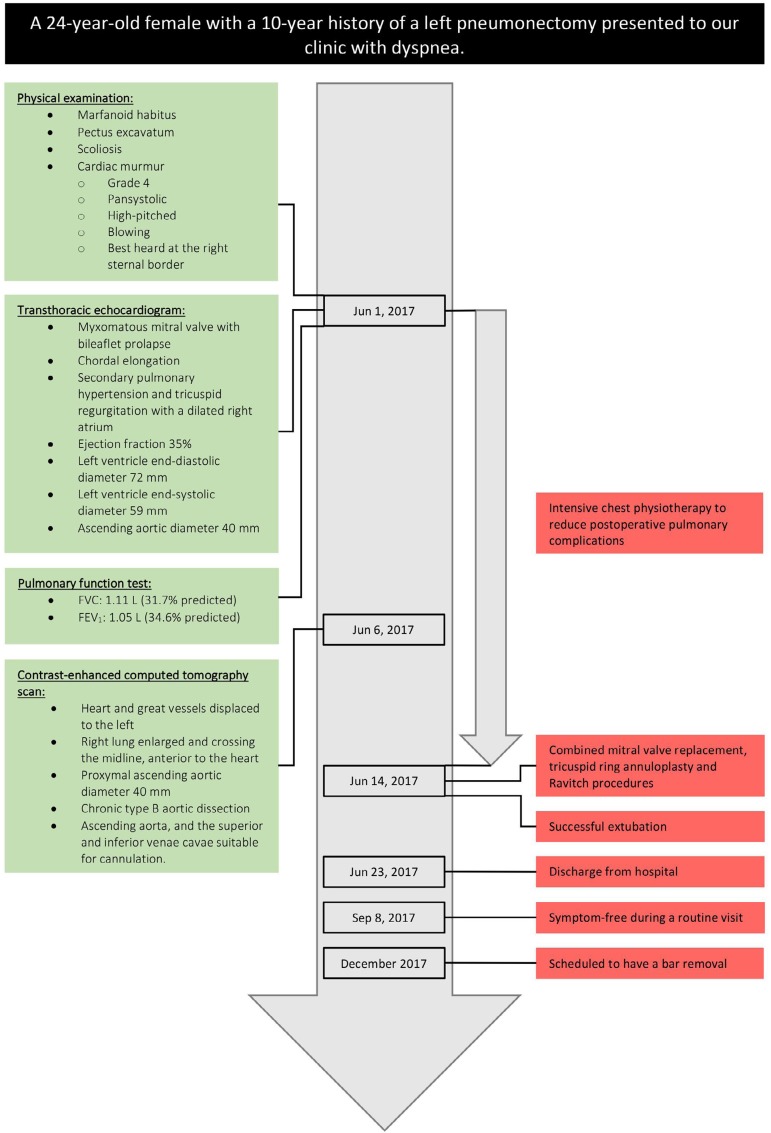
Timeline of interventions and outcomes. FEV_1_=forced expiratory volume in 1^st^ second; FVC=forced vital capacity

The Figure 5 presents a timeline of interventions and outcomes.

## DISCUSSION

After conducting a Medline search from 1966 to April 2018 using the search terms "pneumonectomy" and "open heart surgery" or "coronary artery bypass" or "mitral valve" or "aortic valve" or "revascularization", we identified 30 articles in English language^[3-34]^. A total of 42 cardiac operations were performed on 38 patients, including the current one (Table 1). The mean patient age was 65.2 years (range: 24-83 years). Twenty-one (76.3%) patients were male. There were 20 (47.6%) isolated coronary artery bypass grafting (CABG) procedures, 18 (42.8%) valvular procedures, and 4 (9.5%) combined CABG and valvular procedures. Two of these operations were transapical aortic valve implantation procedures (patients 29 and 30)^[26,27]^.

**Table 1:** Summary of 38 patients with previous pneumonectomy who underwent cardiac surgery.

Patient no.	Author	Publication year	Sex	Age	Pneumonectomy site	Years elapsed after pneumonectomy	Indication for pneumonectomy	Preoperative data		Operation	Operative details	Complications	Length of hospital stay (days)
								FEV_1_ (percent of predicted value)	FVC (percent of predicted value)				
1	Berrizbeitia et al.^[3]^	1994	M	61	Right	42	Bronchiectasis	21	32	CABG	- 3 SVGs to LAD, OMB, and PDA - Median sternotomy - On-pump	None	8
2	Shibata et al.^[4]^	1994	M	67	Left	13	Cancer	77	55	CABG	- 3 SVGs - Median sternotomy - On-pump	None	57
3	Medalion et al.^[5]^	1994	F	70	Left	40	Tuberculosis	45	52	CABG	- LITA and 3 SVGs - Median sternotomy - On-pump	None	11
4	Demirtas et al.^[6]^	1995	M	63	Left	20	Cancer	36	36	CABG	- LITA and SVG to LAD and OMB - Median sternotomy - On-pump	Prolonged inotropic support, low cardiac output requiring intra-aortic balloon pump insertion, right-sided pneumothorax requiring re-intubation and chest tube insertion, mediastinitis and sternal detachment requiring re-operation, sepsis, and death	12
5	Izzat et al.^[7]^	1995	M	65	Right	10	Cancer	N/A	N/A	Mitral valve replacement	- Approach to mitral valve through left atrial appendage - Median sternotomy - On-pump	None	7
6	Soltanian et al.^[8]^	1998	F	70	Left	19	Cancer	N/A	N/A	CABG	- SVG to LAD - Left thoracotomy - Off-pump	None	7
7	Lippmann and Au^[9]^	2000	M	68	Left	15	Cancer	56	60	CABG	- SVGs - Median sternotomy - On-pump	Bronchopneumonia, pulmonary embolism, respiratory failure requiring re-intubation, and death	6
8			M	73	Left	22	Cancer	53	58	CABG	- LITA and SVGs - Median sternotomy - On-pump	Postoperative bleeding requiring re-exploration, atrial fibrillation, hemothorax requiring re-intubation, and chest tube insertion	48
9	Gölbasi et al.^[10]^	2001	M	58	Right	0.75	Cancer	50	44	CABG	- SVGs to LAD, OMB, and RCA - Median sternotomy - On-pump	None	9
10	Diab et al.^[11]^, Jamaleddine and Obeid^[12]^	2001	M	64	Right	6	Trauma	N/A	N/A	CABG	- SVGs to LAD, Cx, and RCA - Median sternotomy - On-pump	Respiratory failure requiring re-intubation	12
11	El-Hamamsy et al.^[13]^	2003	F	65	Right	51	Tuberculosis	36	44	Mitral valve replacement and tricuspid valve annuloplasty	- Standard left atrial approach - Median sternotomy - On-pump	Pneumothorax requiring chest tube insertion	20
12			F	71	Right	50	Tuberculosis	28	27	CABG	- 3 SVGs - Median sternotomy - Off-pump	None	6
13	Kumar et al.^[14]^	2003	M	70	Left	15	Cancer	N/A	N/A	CABG	- LITA and SVG to LAD and PDA - Median sternotomy - Off-pump	None	7
14	Erdil et al.^[15]^	2004	M	51	Right	17	Tuberculosis	45	43	CABG	- 2 RAs to LAD, OMB, and RCA - Median sternotomy - On-pump	None	5
15	Shanker et al.^[^16^]^	2005	M	80	Left	27	Cancer	46	N/A	CABG, mitral valve repair, and aortic valve replacement	- 1 SVG to LAD and diagonal artery - Approach to both valves via aortotomy - Bioprosthetic aortic valve - Median sternotomy - On-pump	None	10
16	Bernet et al.^[17]^	2006	M	58	Right	3	Cancer	59	59	CABG	- LITA and SVG to LAD and OMB - Median sternotomy - Off-pump	None	8
17	Hukusi Us et al.^[18]^	2006	M	74	Left	15	Cancer	45	60	CABG	- LITA and SVG to LAD, Cx, and RCA - Median sternotomy - On-pump	None	7
18	Stoller et al.^[19]^	2007	F	54	Left	3	Cancer	61	61	CABG	- SVGs to LAD and Cx - Left thoracotomy - Off-pump	None	5
19			M	48	Left	18	Cancer	N/A	N/A	CABG	- 3 SVGs to LAD, Cx, and RCA - Median sternotomy - On-pump	Respiratory failure requiring prolonged mechanical ventilation and extracorporeal membrane oxygenation and pneumonia	26
						26		37	42	Mitral and tricuspid valve repair	- Right atriotomy and transseptal approach - Re-sternotomy - On-pump, deep hypothermic circulatory arrest	Atrial fibrillation	13
20			M	71	Left	7	Cancer	33	40	Mitral valve replacement and tricuspid valve annuloplasty	- Right atriotomy and transseptal approach - Median sternotomy - On-pump	Renal failure and atrial fibrillation	N/A
21			F	74	Left	37	Cancer	75	70	CABG	- 4 SVGs to LAD, OMBs, and RCA - Left thoracotomy - On-pump	None	6
22	Sleilaty et al.^[20]^	2007	M	71	Right	20	Trauma	53	48	CABG and aortic valve replacement	- SVG to diagonal artery - Bioprosthetic aortic valve - Median sternotomy - On-pump	None	13
23	Barreda et al.^[21]^	2008	M	68	Left	4	Cancer	N/A	N/A	Aortic valve replacement	- Left anterior thoracotomy - On-pump	Re-exploration for worsening of preoperative mitral insufficiency due to leaflet tethering 1 day after aortic valve replacement	N/A
										Mitral valve annuloplasty	- Left posterior thoracotomy - On-pump		
24	Ghotkar et al.^[22]^	2008	M	71	Left	18	Cancer	42	53	CABG	- SVG to LAD and PDA - Median sternotomy - On-pump	Postoperative bleeding requiring re-exploration and atrial fibrillation	17
25			F	77	Right	1	Cancer	64	63	Aortic valve replacement	- Bioprosthetic aortic valve	None	N/A
26	Zhao et al.^[23]^	2008	M	57	Left	7	Cancer	61.9	70.3	CABG	- 2 SVGs to LAD, RCA, and OMB - Left posterolateral thoracotomy - Off-pump	None	9
27	Us et al. ^[24]^	2010	M	65	Left	8	N/A	45	50	Mitral valve replacement and subaortic membrane resection	- transseptal approach and aortotomy - mechanical mitral valve prosthesis - median sternotomy - on-pump	None	7
28	Stamou et al. ^[25]^	2010	M	83	Left	8	Cancer	48	N/A	CABG and aortic valve replacement	- left anterolateral thoracotomy - on-pump	None	5
29	Ferrari et al. ^[26]^	2011	M	64	Left	8	Cancer	N/A	N/A	Transapical aortic valve implantation	- left anterolateral thoracotomy - off-pump	None	N/A
30	Raja et al. ^[27]^	2011	F	67	Right	18	Cancer	49	N/A	Transapical aortic valve implantation	- right anterior thoracotomy - off-pump	None	4
31	Ushijima et al. ^[28]^	2011	M	82	Left	20	Cancer	63.8	63.8	CABG	- LITA, RA and RGEA to LAD, PL and PDA - left thoracotomy - off-pump	None	N/A
32	Wilhelmi et al. ^[29]^	2013	M	68	Right	8	Cancer	56	58	Aortic valve replacement	- bioprosthetic aortic valve - right anterolateral thoracotomy - on-pump	None	6
33	Dag et al. ^[30]^	2013	M	72	Left	13	Cancer	N/A	N/A	CABG and mitral valve replacement	- SVG to LAD and RCA - standard left atrial approach - mechanical mitral valve prosthesis - median sternotomy - on-pump	None	N/A
34	Gennari et al. ^[31]^	2014	M	71	Left	4	Cancer	53	54	Mitral and tricuspid valve repair	- median sternotomy - on-pump	None	11
35	Rose et al. ^[32]^	2015	M	31	Right	8	Cancer	N/A	N/A	Mitral valve repair	- left atrial approach - video-assisted right thoracotomy - on-pump	None	8
36	Takahashi et al. ^[33]^	2016	M	72	Right	32	Tuberculosis	N/A	N/A	Mitral valve replacement	- Right thoracotomy - on-pump	Periprosthetic leak	N/A
						32		N/A	N/A	Repair of mitral peri-prosthetic leak (2 months after valve replacement)	- Right thoracotomy - on-pump	None	N/A
						40		N/A	N/A	Repair of mitral peri-prosthetic leak (8 years after valve replacement)	- Cranial-sided approach to left atrium - median sternotomy - on-pump	None	N/A
37	Sinha et al. ^[34]^	2016	M	61	Right	47	Scimitar syndrome	N/A	N/A	Mitral valve repair	- left atrial approach - video-assisted right thoracotomy - on-pump	None	5
38	Current patient	2018	F	24	Left	10	Bronchiectasis	34.6	31.7	Mitral valve replacement and tricuspid valve annuloplasty	- standard left atrial approach - median sternotomy combined with Ravitch procedure - on-pump	None	9

CABG=Coronary artery bypass grafting; Cx=circumflex; FEV1=forced expiratory volume in 1st second; FVC=forced vital capacity; LAD=left anterior descending; LITA=left internal thoracic artery; OMB=obtuse marginal branch; PDA=posterior descending artery; RAs=radial arteries; RCA=right coronary artery; SVG=saphenous vein graft

Fifteen (39.4%) patients had a previous right pneumonectomy. The most common indication for pneumonectomy was cancer (n=27, 71%), followed by tuberculosis (n=5, 13.1%), trauma (n=2, 5.2%), bronchiectasis (n=2, 5.2%), scimitar syndrome (n=1, 2.6%), and unknown etiology (n=1, 2.6%). Preoperative FEV_1_ values were available for 28 patients and averaged 49% of predicted (range: 21-77%). Preoperative FVC values were available for 25 patients and averaged 49.2% of predicted (range: 27-70.3%).

The preferred surgical incision was a median sternotomy in 26 (61.9%) cases, a left thoracotomy in 9 (21.4%) cases, a right thoracotomy in 6 (14.2%) cases, and it was not specified in 1 (2.3%) case. Patients 35 and 37 underwent surgery utilizing video-assisted right thoracotomy^[32,34]^. Among 24 CABG operations, a left internal thoracic artery was used as a bypass conduit in 7 (29.1%) cases. The use of a right internal thoracic artery was not reported. Complete arterial revascularization was performed in 2 (8.3%) cases. Among 20 isolated CABG operations, 7 (35%) were carried out without the use of CPB.

Length of hospital stay data was available in 32 cases and averaged 12 days (range: 4-57 days). Postoperative complications were experienced after 11 (26.1%) operations. The most common complication was atrial fibrillation (n=5, 11.9%), followed by respiratory failure requiring re-intubation (n=4, 9.5%), pneumothorax (n=2, 4.7%), pneumonia (n=2, 4.7%), and bleeding requiring re-exploration (n=2, 4.7%). Two (5.2%) patients did not survive to discharge.

Previous pneumonectomy adds two major risks to cardiac operations: (1) there is an increased risk of postoperative pulmonary complications due to reduced lung capacity; (2) heart and great vessels are displaced and rotated, making surgical exposure more difficult.

Six months after pneumonectomy, FVC decreases by 36% and FEV_1_ by 34%. These parameters do not significantly improve beyond 6 months^[2]^. Considering that the pulmonary function may deteriorate significantly after cardiac surgery even in patients who have normal preoperative respiratory function, previous pneumonectomy poses a great risk of postoperative pulmonary complications^[35]^. Hulzebos et al.^[36]^ found preoperative inspiratory muscle training to be effective in preventing postoperative pulmonary complications in high-risk patients undergoing elective CABG surgery. Conventional measures such as avoidance of phrenic nerve injury and fluid overload, early extubation, early mobilization, and postoperative chest physiotherapy should be utilized. Central venous line should be placed on the side of the pneumonectomy to avoid pneumothorax.

Considerable anatomical changes occur in long-term survivors after pneumonectomy. Smulders et al.^[1]^ evaluated the function and position of the heart using dynamic magnetic resonance imaging (MRI) in 15 patients who underwent pneumonectomy at least 5 years ago. They reported that although varying degrees of mediastinal shift occur in all patients, right-sided pneumonectomy is mostly associated with a lateral shift and only a minor rotation, whereas left-sided pneumonectomy leads to a greater degree of rotation^[1]^. Whether the patient had a left or right pneumonectomy, it affects the choice of surgical approach. For instance, in the case of a previous left pneumonectomy, it may be easier to bypass left-sided coronary arteries through a left thoracotomy, rather than a median sternotomy, and mitral and tricuspid valves may be inaccessible from the usual right thoracotomy. Stoller et al.^[19]^ reported difficult exposure of the mitral valve through a median sternotomy in a patient who underwent a left pneumonectomy 9 years ago. However, we found it relatively easy to perform a mitral valve surgery in a similar setting. Because long-term anatomical changes after pneumonectomy vary considerably among patients, preoperative CT and/or MRI should be performed to assess the exact locations of cardiac structures and cannulation sites^[37]^. Decision of surgical approach should only be made after carefully examining the extent of the shift and the rotation of the cardiac structures.

Another subject that needs addressing is the concomitant pectus excavatum. Schmidt et al.^[38]^ advocate simultaneous correction of the pectus excavatum in patients requiring cardiac surgery. We resected deformed cartilages prior to sternotomy to improve surgical exposure as previously reported by Sacco-Casamassima et al.^[39]^.

Cardiac operations on patients with previous pneumonectomy can be performed with a favourable outcome. Thorough preoperative evaluation with imaging studies to assess cardiac position and function and intensive respiratory physiotherapy are essential.

**Table:** 

**Authors’ roles & responsibilities**	
IK	Substantial contributions to the conception or design of the work; or the acquisition, analysis, or interpretation of data for the work; drafting the work or revising it critically for important intellectual content; final approval of the version to be published
ACT	Substantial contributions to the conception or design of the work; final approval of the version to be published
KO	Substantial contributions to the conception or design of the work; final approval of the version to be published
YO	Substantial contributions to the conception or design of the work; final approval of the version to be published
AB	Substantial contributions to the conception or design of the work; final approval of the version to be published
MY	Substantial contributions to the conception or design of the work; final approval of the version to be published
